# Malocclusion Pattern (Angle's) in Mauritian Orthodontic Patients

**DOI:** 10.5402/2012/210306

**Published:** 2012-03-28

**Authors:** B. H. Durgesh, Prashanth Prakash, Ravikumar Ramakrishnaiah, Basavaraj Subashchandra Phulari, Abdul Aziz A. Al Kheraif

**Affiliations:** ^1^Faculty, Dental Health Department, Dental Biomaterials Research Chair, College of Applied Medical Sciences, King Saud University, Riyadh 11433, Saudi Arabia; ^2^Department of Pedodontics, MS Ramaiah Dental College and Hospital, Bangalore, Karnataka, India; ^3^Dental Biomaterials Research Chair, College of Applied Medical Sciences, King Saud University, Riyadh 560013, Saudi Arabia; ^4^Faculty, Department of Orthodontics, Mauras, College of Dentistry and Hospital, Arsenal, Mauritius

## Abstract

The aim of the study was to assess the pattern of malocclusion in different ethnic group of Mauritian population visiting the Orthodontic Department at Mauras College of Dentistry and Hospital, Republic of Mauritius. The study population comprised of 624 patients who visited the orthodontic department during 2010. The clinical examination was conducted by a well-calibrated orthodontist. The data were recorded in the case sheets and was analyzed for presence of angles class I, class II, and class III malocclusion in both male and female patients of Asian, African, Caucasian, and Chinese ethnicity aged 5–55 years. Malocclusion was found to be high in females compared to males. 414 patients (150 male + 264 female) presented with class I, 182 patients (52 male + 130 female) presented with class II, and 28 patients (12 male + 16 female) presented with class III. Asian ethnic group were more affected and patient seeking orthodontic treatment was high in 11–15 years age group.

## 1. Introduction

Mauritius is a sparkling crystal in the turquoise waters of the Indian ocean. The island has maintained one of the developing world's most successful democracies and has enjoyed years of constitutional order and also the most developed of the Mascarene Islands; the contrast of colors, cultures, and tastes makes the island very charming. Mauras College of Dentistry and Hospital is the only fully functional college in the island providing good service in dental health care sector. There is hardly any study on pattern of malocclusion done on the Mauritian patients seeking orthodontic treatment. So this study aims at understanding the level of malocclusion present and evaluates the treatment need in different age group, sex, and ethnicity of Mauritian orthodontic patients.

The epidemiological data on the prevalence of malocclusion is an important determinant in planning appropriate levels of orthodontic treatment [1]. A large number of epidemiological studies have been carried out to determine the prevalence of malocclusion in different racial and ethnic groups and the reported incidences varied in different populations [2–6]. The Angle's classification method has been widely used as a qualitative epidemiological tool for malocclusion assessment [7].

## 2. Materials and Methods

The orthodontic records of 624 patients who attended the Department of Orthodontics at Mauras College of Dentistry were taken as the study population.

Patients with a history of previous orthodontic treatment or with systemic disease, craniofacial deformities, or syndrome and patients with incomplete records were excluded from this study. All patients were self-referred. Data was collected in case sheets by an orthodontist at the Department of Orthodontics. Examinations were carried out using a mirror and probe and a pen light wielded by an assistant.

The criteria of examination included qualitative method of recording occlusal traits, that is, Angle's classification to determine the anterior-posterior dental arch relationship [8]. This method is useful for easy documentation and provides a common channel of communication among dental professionals. The readings taken either from the first permanent molar relationship, or in the case of its absence or extraction, the canine relationship was marked. Angles class II divisions I and II were not segregated but considered as fully class II. 

Data analysis was performed using SPSS statistical software for Windows v.13.0 (SPSS Inc., Chicago, IL, USA).

## 3. Results

The study demonstrated the significant difference in distribution of malocclusion (Table 1) between males (34.3%) and females (65.7%).

Asian males and females had highest distribution of class I malocclusion with 87.9% and least with Chinese 1.1% (Table 2).

The distribution of class II malocclusion in Asian and Africans females (Table 3) was highly significant (*P* < 0.01).

Class III malocclusion was highly significant (*P* < 0.01) in African females (Table 4). Malocclusion in relation to different age group showed highly significant values in 11–15 years age group with class I and class II malocclusion and 16–20 years age group with class III malocclusion (Table 5).

## 4. Discussion

The evaluation of orthodontic patients with many variables (age, sex, and ethnicity) may give valuable information for planning orthodontic treatment. According to the evaluation Angle class I malocclusion was considered the most prevalent type of malocclusion with 66.3% followed by 29.2% class II and 4.5% class III among the orthodontic patients examined.

The predominance of Angle's class I malocclusion in this study may also be attributed to sample selection and ethnic variation.

Distribution of different type of malocclusion may show great variability even in a population of same origin [9]. Although angle's classification is limited in that it does not incorporate vertical and transversal abnormalities, it is a universally accepted system that is reliable and repeatable and that minimizes examiner subjectivity [10]. The present study confirmed that predominant anterior posterior relationship of the arches in examined subjects was class I malocclusion with significant gender differences. The male: female ratio in our study was 1 : 2, which is similar to other studies [9, 11]. Female study group presented with high number of class II and class III malocclusion. On the other hand, Onyeaso et al. [12] reported that males were found to have significantly more of classes II and III molar relationships than females.

## 5. Conclusion

This study revealed the predominance of class I malocclusion among the orthodontic patients and Asians were the most common with malocclusion as compared with Africans, Caucasians, and Chinese.

 Treatment need was high in 11–15 years age group, the reason being the esthetic concern of that particular age group. The study also indicated the scope of adult orthodontics as the study population had good number of patients seeking orthodontic treatment above 40 years of age.

## Figures and Tables

**Table 1:** Distribution of Angle's malocclusion according to sex.

	Class I	Class II	Class III
	*n*	*n*	*n*
Male (m)	150 (36)	52 (28.6)	12 (43)
Female (f)	264 (64)*	130 (71.4)**	16 (57)

Sum (m + f)	414 (100)	182 (100)	28 (100)

*P < 0.05, **P < 0.01.

**Table 2:** Distribution of Angle's class I malocclusion in male and female of different ethnicity.

	Asian	African	Caucassian	Chinese
	*n*	*n*	*n*	*n*
Male (m)	142 (39)	6 (15)	0 (0)	2 (50)
Female (f)	222 (61)*	34 (85)**	6 (100)	2 (50)

Sum (m + f)	364 (100)	40 (100)	6 (100)	4 (100)

*P < 0.05, **P < 0.01.

**Table 3:** Distribution of Angle's class II malocclusion in male and female of different ethnicity.

	Asian	African	Caucassian	Chinese
	*n*	*n*	*n*	*n*
Male (m)	48 (28)	2 (25)	2 (100)	0 (0)
Female (f)	122 (72)**	8 (75)**	0 (0)	0 (0)

Sum (m + f)	170 (100)	10 (100)	2 (100)	0 (0)

*P < 0.05, **P < 0.01.

**Table 4:** Distribution of Angle's class III malocclusion in male and female of different ethnicity.

	Asian	African	Caucassian	Chinese
	*n*	*n*	*n*	*n*
Male (m)	10 (55.5)	2 (25)	0 (0)	0 (0)
Female (f)	8 (44.5)	8 (75)**	0 (0)	0 (0)

Sum (m + f)	18 (100)	10 (100)	0 (0)	0 (0)

*P < 0.05, **P < 0.01.

**Table 5:** Distribution of Angle's classification according to different age group.

	Class I	Class II	Class III
Age (years)	*n*	*n*	*n*
5–10	56 (13)	28 (15.4)	0 (0)
11–15	170 (41)*	88 (48.4)*	8 (29)
16–20	90 (22)	34 (18.7)	12 (43)*
21–25	48 (12)	14 (7.7)	4 (14)
26–30	26 (6)	8 (4.4)	2 (07)
31–35	8 (2)	4 (2.2)	0 (0)
36–40	6 (1.5)	4 (2.2)	2 (07)
41–45	4 (1)	0 (0)	0 (0)
46–50	4 (1)	2 (1.1)	0 (0)
51–55	2 (0.5)	0 (0)	0 (0)

Sum	414 (100)	182 (100)	28 (100)

*P < 0.05, **P < 0.01.
